# Characterization of 15-*cis*-ζ-Carotene Isomerase Z-ISO in Cultivated and Wild Tomato Species Differing in Ripe Fruit Pigmentation

**DOI:** 10.3390/plants10112365

**Published:** 2021-11-02

**Authors:** Gleb I. Efremov, Anna V. Shchennikova, Elena Z. Kochieva

**Affiliations:** Research Center of Biotechnology, Institute of Bioengineering, Russian Academy of Sciences, Leninsky Ave. 33, Bld. 2, Moscow 119071, Russia; shchennikova@yandex.ru (A.V.S.); ekochieva@yandex.ru (E.Z.K.)

**Keywords:** carotenogenesis, fruit color, *Solanum* section Lycopersicon, 15-*cis*-ζ-carotene isomerase Z-ISO, promoter analysis, SNPs

## Abstract

Isomerization of 9,15,9′-tri-*cis*-ζ-carotene mediated by 15-*cis*-ζ-carotene isomerase Z-ISO is a critical step in the biosynthesis of carotenoids, which define fruit color. The tomato clade (*Solanum* section Lycopersicon) comprises the cultivated tomato (*Solanum lycopersicum*) and 12 related wild species differing in fruit color and, thus, represents a good model for studying carotenogenesis in fleshy fruit. In this study, we identified homologous *Z-ISO* genes, including 5′-UTRs and promoter regions, in 12 *S. lycopersicum* cultivars and 5 wild tomato species (red-fruited *Solanum pimpinellifolium*, yellow-fruited *Solanum cheesmaniae*, and green-fruited *Solanum chilense*, *Solanum habrochaites*, and *Solanum pennellii*). *Z-ISO* homologs had a highly conserved structure, suggesting that Z-ISO performs a similar function in tomato species despite the difference in their fruit color. *Z-ISO* transcription levels positively correlated with the carotenoid content in ripe fruit of the tomatoes. An analysis of the *Z-ISO* promoter and 5′-UTR sequences revealed over 130 *cis*-regulatory elements involved in response to light, stresses, and hormones, and in the binding of transcription factors. Green- and red/yellow-fruited *Solanum* species differed in the number and position of *cis*-elements, indicating changes in the transcriptional regulation of *Z-ISO* expression during tomato evolution, which likely contribute to the difference in fruit color.

## 1. Introduction

Carotenoids are yellow, orange, and red pigments that are essential for plant development and survival, and that play an important role in healthy human nutrition [1,2]. In plants, the biosynthesis of carotenoids occurs in plastids; it starts with the formation of colorless 15-*cis*-phytoene from two molecules of geranylgeranyl-diphosphate, which is mediated by phytoene synthase (PSY) (Supplementary Figure S1). The following sequential reactions catalyzed by phytoene desaturase (PDS), ζ-carotene desaturase (ZDS), and two carotene *cis*-trans isomerases (CRTISO) yield trans-lycopene—a red pigment serving as a precursor for the synthesis of orange α- and β-carotenes, which then can be converted to yellow lutein and xanthophyll, respectively [3,4,5,6]. A key step in carotenoid biosynthesis is the *cis*-to-*trans* isomerization of the 15–15′ C=C bond in 9,15,9′-tri-*cis*-ζ-carotene synthesized by PDS, which yields 9,9′-di-*cis*-ζ-carotene—a substrate for ZDS [7]. In photosynthetic tissues, this isomerization is partially mediated by light; however, enzymatic catalysis by the integral membrane protein 15-*cis*-ζ-carotene isomerase (Z-ISO) is essential, especially in nonphotosynthetic tissues [6,7,8]). The catalytic function of Z-ISO, which belongs to the nitrite and nitric oxide reductase U (NnrU) family, requires heme cofactor b that, in the reduced state, triggers conformational changes in the active site of the enzyme to allow substrate binding [6]. Such dependence of Z-ISO activity on the redox status of the plastids, which is subjected to changes in response to various internal and external signals, makes Z-ISO a key enzyme in the dynamic control of carotenoid biosynthesis and flux [6,9,10,11]. Z-ISO plays a critical role in carotenoid production not only in the absence of light exposure [6,7,10], but also at temperature fluctuations [12,13], indicating possible involvement of this enzyme in the evolutionary adaptation of plants to environmental changes [6] and in the breeding of cultivars with consolidated valuable traits. 

The *Z-ISO* gene is present in the genomes of all oxygenic phototrophs but absent in anoxygenic species such as green sulfur bacteria and *Chloracidobacterium* [14]. However, to date Z-ISO homologs have been characterized only in cyanobacterium *Arthrospira platensis*, eukaryotic microalga *Euglena gracilis*, and three species of higher plants—*Arabidopsis thaliana*, *Zea mays*, and *Oryza sativa* [5,6,7,8,14,15]. These studies indicate that the inhibition of *Z-ISO* expression blocks the production of carotenoids and end-products of the carotenoid pathway (strigolactones and abscisic acid [ABA]), resulting in abnormal chloroplast development, reduced chlorophyll levels, and decreased photosynthesis [5,7,8,10,15]. In *O. sativa*, *htd12* and *mit1* mutations in the *Z-ISO* gene result in the high-tillering and dwarf phenotypes; the *htd12* plants light green leaves and has an albino phenotype in the dark [5,8]. In *Z. mays* and *A. thaliana*, *Z-ISO* mutations *y9* and *zic1*, respectively, lead to the accumulation of *cis*-carotenes 9,15,9′-tri-*cis*-z-carotene, phytofluene, and phytoene in etiolated tissues and to the appearance of leaves with transverse pale green stripe in *Z. mays* and cotyledons with a significant delay of chlorophyll accumulation in *A. thaliana* [10,16]. Furthermore, it has been shown that in *A. thaliana* an epistatic interaction between *z-iso-155* and *carotenoid chloroplast regulation 2* (*ccr2*) mutations blocks the biosynthesis of specific *cis*-carotenes but restores the formation of prolamellar bodies abrogated by *ccr2* [16]. However, the role of Z-ISO in carotenoid accumulation and coloration of fruit has not been yet investigated.

The tomato clade (*Solanum* section Lycopersicon) comprises the cultivated tomato *Solanum lycopersicum* and related wild species differing in ripe fruit color. Thus, tomatoes represent a good model with which to study carotenogenesis in fleshy fruit and its association with fruit color. In this study, we identified and characterized *Z-ISO* homologs in 12 *S. lycopersicum* cultivars and five wild tomato species in terms of structure, polymorphism, transcriptional regulatory elements, and expression pattern in photosynthetic and nonphotosynthetic tissues. 

## 2. Results

### 2.1. Identification and Structural and Phylogenetic Characterization of Z-ISO Homologs in Tomato Cultivars and Wild Species

**Figure 1:**
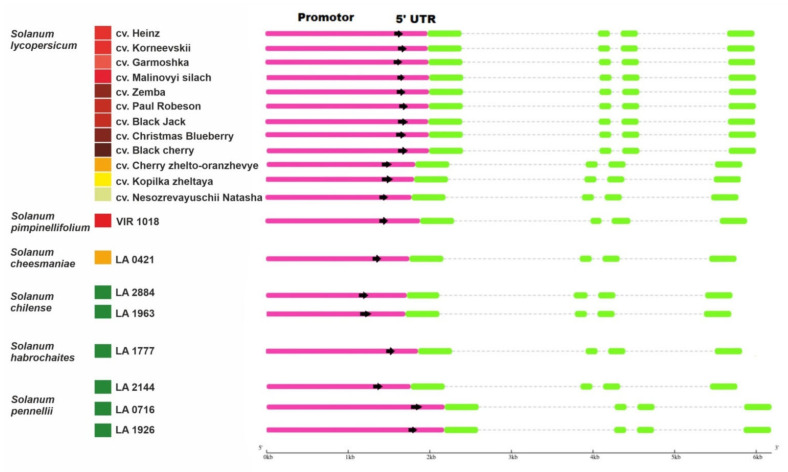
Structures of the identified *Z-ISO* homologous genes in *Solanum lycopersicum* cultivars and wild tomato species (*Solanum* section Lycopersicon). Boxes indicate promoters/5′-UTRs (pink) and exons (green). The colored squares on the left edge of the picture indicate the color of the ripe fruit.

A total of 21 complete sequences of *Z-ISO* homologous genes, including 5′-UTR and promoter regions, were amplified from 12 *S. lycopersicum* cultivars, differing in fruit color, and eight accessions of five wild tomato species: red-fruited (RF) *Solanum pimpinellifolium*, yellow-fruited (YF) *Solanum cheesmaniae*, and green-fruited (GF) *Solanum chilense*, *Solanum habrochaites*, and *Solanum pennellii*; their GenBank accession numbers are listed in Table 1 and Supplementary Table S2). In the genome of *S. lycopersicum* cv. Heinz 1706, the *Z-ISO* gene is located on chromosome 12 (NC 015449.3: 67104216–67112934). The size of the identified *Z-ISO* genes ranged from 3972 to 4010 bp because of intron variability, whereas the coding sequences (CDSs) were of the same size (1110 bp) in all the analyzed accessions and consisted of four exons (Table 1, Figure 1). The fragment upstream of the start codon, which included the promoter and 5′-UTR (1716–2182 bp, depending on the accession) was amplified by PCR and sequenced (Table 1, Supplementary Table S2). 

Compared to the *Z-ISO* gene of *S. lycopersicum* cv. Heinz 1706 (NC 015449.3), 603 single nucleotide polymorphisms (SNPs) were identified in complete *Z-ISO* homologous genes (14.89% variability): 115 SNPs in exons (10.36%), 298 in introns (12.2%), 115 in 5′-UTRs (21.50%), and 419 in promoters (17.14%). Exons I, II, III, and IV contained 50, 16, 24, and 25 SNPs, respectively. Among the 50 SNPs of exon I, 11 were cultivar-specific, 36 were detected only in wild species, and 3 (including 2 nonsynonymous SNPs leading to neutral amino acid [aa] substitutions K20R and R59G) were found in cultivars Korneevskii, Garmoshka, and Zemba, and in wild species *S. pimpinellifolium*, *S. cheesmaniae*, *S. pennellii* (LA0716 and LA1926), *S. habrochaites* (LA1777), and *S. chilense* (LA2884). In exons II/III/IV, 11/3/16 and 5/21/9 SNPs were specific to the *Z-ISO* genes of the cultivars and wild species, respectively. Compared to the *Z-ISO* CDS of RF cv. Heinz 1706, those of RF *S. pimpinellifolium* (LA0480 and VIR1018) and YF *S. cheesmaniae* (LA0421) contained 1, 15, and 2 SNPs, respectively.

Table 2 presents the variability of *Z-ISO* homologs in cultivars, wild species, RF accessions (including *S. cheesmaniae*), GF accessions vs. cv. Heinz1706, and in GF accessions vs. *S. arcanum* LA2157.

**Figure 2:**
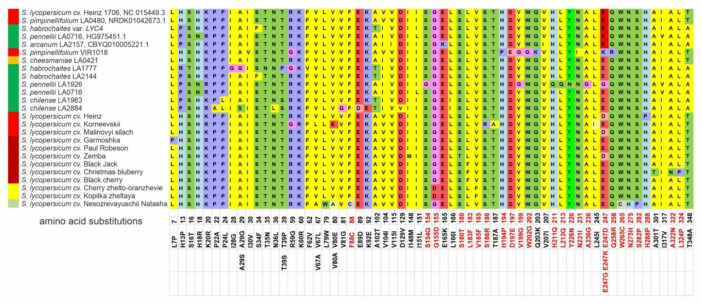
Polymorphisms in tomato Z-ISO homologs. The numbers indicate the positions of single nucleotide polymorphisms (SNPs) in the *Z-ISO* coding sequences (exons I–IV) relative to *S. lycopersicum Z-ISO*; the resulting aa substitutions in the translated Z-ISO proteins are shown below. Nonsynonymous SNPs and PROVEAN-predicted radical aa substitutions are marked red. Ripe fruit color is indicated to the left of the accession name.

Comparative analyses of the translated Z-ISO homologs revealed that 64 out of 115 SNPs led to aa substitutions; among them, three nonsynonymous SNPs led to neutral aa substitutions in wild GF and YF species: V104I (GF and YF); H13P/R and T348A (only GF) (Figure 2). 

The average polymorphism level of *Z-ISO* genes in the analyzed accessions was 16.80% (Table 2). Out of 30, 10, 15, and 10 nonsynonymous SNPs in exons I–IV, 7, 7, 2, and 7 substitutions were specific to cultivars and 21, 3, 13, and 3—to wild species, respectively. The largest number of aa substitutions were detected in Z-ISO of GF species *S. pennellii* LA1926, *S. chilense* LA2884, and *S. habrochaites* LA1777; among RF/YF accessions, Z-ISO of *S. pimpinellifolium* VIR1018 was the most variable (Figure 2). Among the cultivars, the largest number of aa substitutions were found in the Z-ISO of cv. Korneevskii (Figure 2). 

According to PROVEAN, 26 of 63 aa substitutions were predicted to be radical; most of them were detected in exon III (14), followed by exons II (6), IV (5), and I (1) (Figure S1). In wild species, radical substitutions were mostly accession-specific (such as S154G, H211Q, L213Q, Y226N, A236G, and E247G in *S. pennellii* LA1926, or H194P, D197E, W202G, and E247K in *S. pimpinellifolium* VIR1018). In cultivars, radical mutations were specific to individual cultivars (for example, F88C, W265C, N275H, and S282P—to cv. NN-25, A322N and L324P—to cv. Christmas blueberry, S180T—to cv. Zemba, or S186R—to cv. Korneevskii) as well as to several cultivars (for example, E247D—to cv. Heinz, Malinovyi silach, and Zemba, G155D—to cv. Kopilka zheltaya and Cherry zhelto-oranzhevye, or L183F—to cv. Black Jack, Christmas blueberry, and Black cherry) (Figure 2). Residues H153, H269, C266, and D297, which have been shown to be important for Z-ISO enzymatic activity as potential heme or iron axial ligands [17], were conserved among the analyzed accessions (Supplementary Figure S3).

According to UniProt prediction for *A. thaliana* Z-ISO, all translated Z-ISO homologs consisted of an N-terminal transit peptide (1–58 aa) carrying no radical aa substitutions and a mature protein corresponding to the Z-ISO enzyme (59–369 aa) (Figure 2). According to the NCBI Conserved Domains Database (CDD) search, the Z-ISO homologs contained the COG4094 domain (141–369 aa) characteristic for membrane proteins of the NnrU superfamily, and according to SignalP and ChloroP prediction servers, a transit peptide could be located within the 1–70/80 aa region.

A three-dimensional (3D) model of tomato Z-ISO homologs was constructed based on crystal structures of isoprenylcysteine carboxyl methyltransferase from *Methanosarcina acetivorans* (140–319 aa [48% coverage], reliable identity [98.8%]; PDB: 4A2N; https://doi.org/10.2210/pdb4A2N/pdb, accessed on 18 October 2021), *Saccharomyces cerevisiae* membrane protein (86–156 aa [18% coverage], low identity [20.7%]; PDB: 2K9P; https://doi.org/10.2210/pdb2K9P/pdb, accessed on 18 October 2021), and papillomavirus E1 hexameric helicase (15–65 aa [13% coverage], low identity [18%]; PDB: 2GXA; https://doi.org/10.2210/pdb2GXA/pdb, accessed on 18 October 2021). Overall, we could model 49% of residues (a part of the Z-ISO catalytic domain) with more than 90% confidence; 189 residues were modeled ab initio (Supplementary Figure S4).

The Z-ISO enzyme had a helical topology, including transmembrane helices predicted with high confidence: S1 (97–116 aa), S2 (139–157 aa), S3 (172–191 aa), S4 (210–234 aa), S5 (258–273 aa), S6 (277–294 aa), and S7 (340–363 aa) (Supplementary Figure S4). Cytosol-located regions (116–139 aa, 191–210 aa, 273–277 aa, and 363 aa–C-terminus) may form the cofactor-binding pocket positioned at the base of the transmembrane helices. Similar results were obtained for the most variable Z-ISO homologs from GF *S. pennellii* LA1926, *S. chilense* LA2884, and *S. habrochaites* LA1777. No species- or fruit color-specific structural features were observed. 

**Figure 3:**
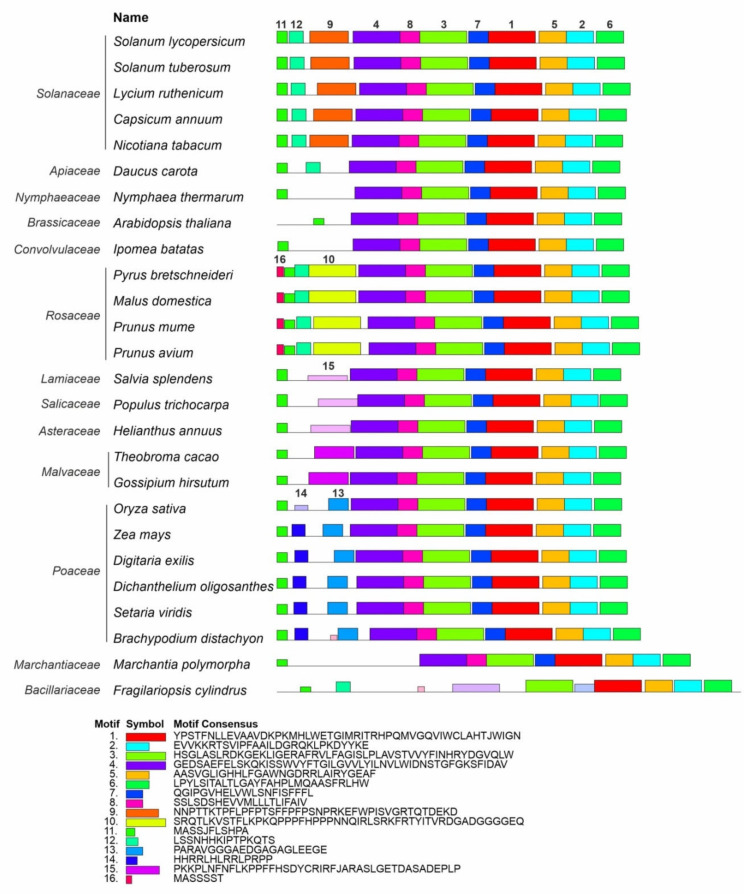
Distribution of conserved motifs in Z-ISO homologs of different plant species. Identification was conducted using the MEME search tool; the length of each box corresponds to that of the motif and the order of the motifs corresponds to their position in the protein. Motifs are numbered in descending order of the reliability of their conservatism.

A MEME-based analysis of the Z-ISO homologs identified in this study and 58 Z-ISO proteins of different plant species extracted from the NCBI database (Supplementary Table S1) revealed 16 reliable conserved motifs. Among them, eight motifs (1–7, and 11) were common for all Z-ISO homologs and the other eight were specific to individual genera or families (motif 8—to all analyzed species excluding diatoms, motif 9—to Solanaceae, motifs 10 and 16—to Rosaceae, motifs 13 and 14—to Poaceae, and motif 15—to Malvaceae, Asteraceae, Salicaceae, and Lamiaceae; a motif 16-like consensus was also found in *Brachypodium distachyon* and unicellular diatom microalgae *Fragilariopsis cylindrus*). We observed at least four different consensus patterns in the N-terminal transit peptide, all of which contained motif 11 consisting of two short α-helices (according to Phyre2; Supplementary Figure S4): motifs 11, 12, and 9—in Solanaceae, motifs 16, 11, 12, and 10—in Rosaceae, motifs 11 and 15—in Malvaceae, Asteraceae, Salicaceae, and Lamiaceae, and motifs 11, 14, and 13—in Poaceae (Figure 3). 

**Figure 4:**
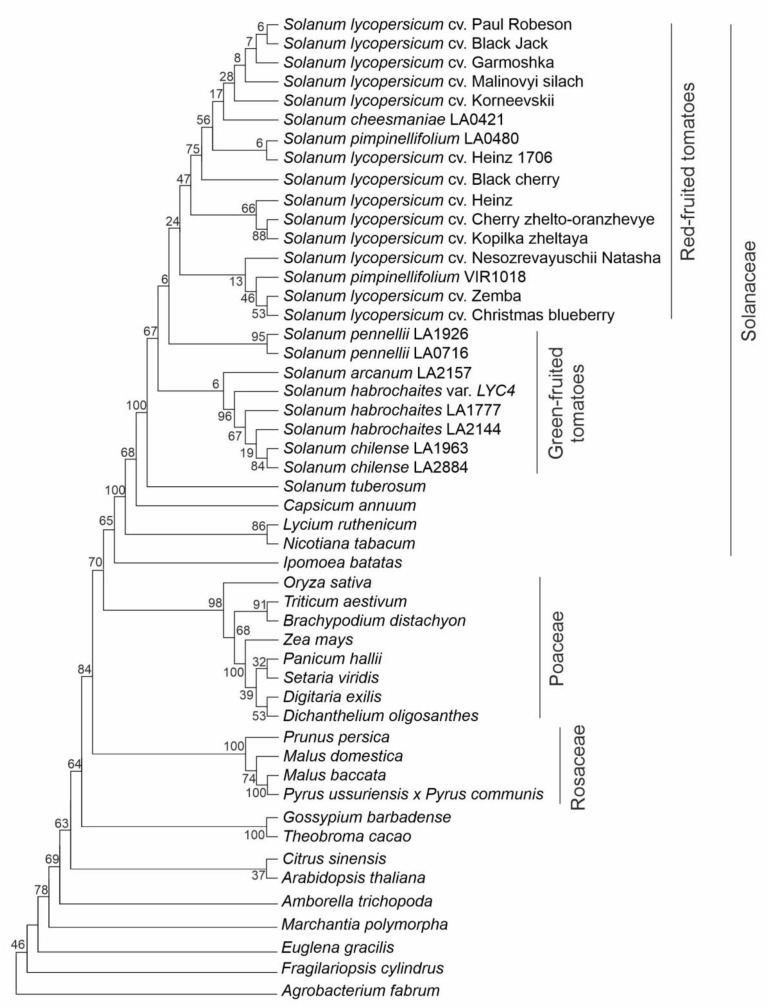
Evolutionary relationships among plant species based on Z-ISO protein sequences. *Agrobacterium* NnrU protein was used as an outgroup sequence. Analysis was performed using the Maximum Likelihood method based on the Jones-Taylor-Thornton (JTT) matrix-based model; initial tree(s) for the heuristic search were obtained automatically by applying neighbor-joining (NJ) and BioNJ algorithms to a matrix of pairwise distances estimated using a JTT model; then, a topology with a superior log likelihood value was selected. A discrete Gamma distribution was used to model evolutionary rate differences among the sites (2 categories, +G, parameter = 0.6496). Percentages of replicate trees in which the associated taxa clustered together in the bootstrap test (1000 replicates) are shown next to the branches.

A phylogenetic analysis of putative Z-ISO protein sequences identified in this study and extracted from the database (Supplementary Table S1) revealed five main clades corresponding to Solanaceae, Poaceae, and Rosaceae (Figure 4). The Solanaceae clade had *Nicotiana* and *Lycium* species at the base, and the *Solanum* cluster with *S. tuberosum* positioned at the base was the most evolutionary recent. Tomato species were divided into two main subclades comprising RF and GF species, respectively (Figure 4). Poaceae plants are grouped together. The Rosaceae species (Fabids), as expected, formed sister cluster to Malvids, which consists of two subclusters: (1) *Gossipium barbadense* and *Theobroma cacao* (Malvales); (2) *Citrus sinensis* (Sapindales) and *A. thaliana* (Brassicales) (Figure 4). The unification of *C. sinensis* and *A. thaliana* belonging to different orders may be due to the absence of other representatives of these two orders in the analysis.

### 2.2. In Silico Analysis of S. lycopersicum Z-ISO Expression 

The expression of *Z-ISO* genes in various tissues (roots, leaves, buds, flowers, and fruit) of *S. lycopersicum* cv. Heinz 1706, in parts of *S. lycopersicum* cv. M82 ripe fruit, and in *S. pimpinellifolium* LA1589 seeds was assessed based on transcriptomics data (TomExpress and Tomato Expression Atlas). As the fruit is the organ most enriched in carotenoids, analysis of its gene expression was more detailed than that of the other tissues and included 11 fruit parts (from the outer epidermis to seeds) at 16 developmental stages (from anthesis to red ripe).

**Figure 5:**
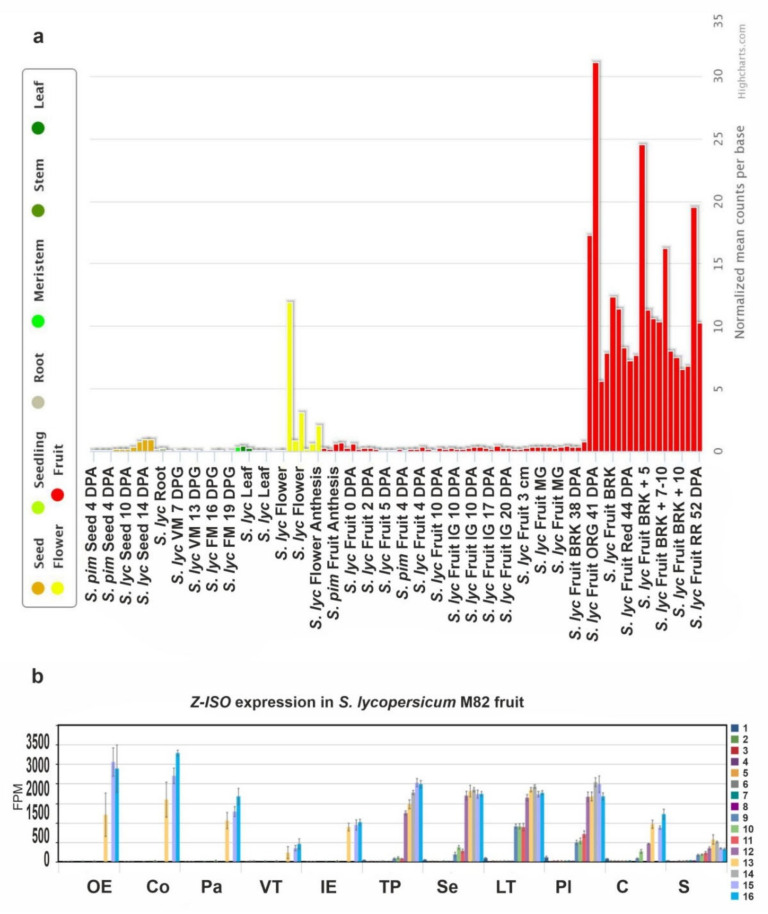
The *Z-ISO* expression pattern in tomato. (**a**) Diagram of *Z-ISO* mRNA expression in wild type *S. lycopersicum* (*S. lyc*) and *S. pimpinellifolium* (*S. pim*) (according to the TomExpress database, http://tomexpress.toulouse.inra.fr/, accessed on 10 August 2021). Gene transcription was analyzed in roots, leaves, meristems (vegetative meristem, VM; floral meristem, FM), flowers, fruit (immature green, IG; mature green, MG; breaker, BRK; orange, ORG; red ripe, RR), and seeds. DPA, days postanthesis; DPG, days postgermination; the numbers after “+” indicate number of days. (**b**) Diagram of *Z-ISO* mRNA expression in different *S. lycopersicum* cv. M82 fruit tissues (OE—outer epidermis; Co—collenchyma; Pa—parenchyma; VT—vascular tissue; IE—inner epidermis; TP—total pericarp; Se—septum; LT—locular tissue; Pl—placenta; C—columella; S—seeds) at 16 stages (1—anthesis; 2—5 DPA; 3—10 DPA; 4—20 DPA; 5—30 DPA; 6—MG stem; 7—MG equatorial; 8—MG stylar; 9—BRK stem; 10—BRK equatorial; 11—BRK stylar; 12—Pink stem; 13—Pink equatorial; 14—Pink stylar; 15—Light Red; 16—Red Ripe) of growth and maturation (according to https://tea.solgenomics.net, accessed on 10 August 2021).

Figure 5a shows that *Z-ISO* was expressed in all plant tissues, with the maximum level in the breaker and fully ripe fruit, followed by the flower, and the seed at 14 days postanthesis. In the fruit, the expression of *Z-ISO* was upregulated with the growth and maturation, reaching the highest level at the fully ripe stage (pink, light red, and red fruit) with the minimum in seeds and vascular tissue (Figure 5b). 

### 2.3. Analysis of Z-ISO Promoters

Next, we analyzed the presence of *cis*-regulatory elements in *S. lycopersicum* cv. Heinz 1706 (FSCV) and *S. pennellii* LA0716 *Z-ISO* sequences upstream of the initiation codon (~2.0 kb), which predictably included the 5′-UTR (363–473 bp, Table 1) and promoter (1.5–1.6 kb). The length of the regulatory region was chosen based on the size of the predicted *A. thaliana Z-ISO* (AT1G10830.1) gene promoter (1.2 kb; available in the Plant Promoter Database; http://ppdb.agr.gifu-u.ac.jp; accessed on 28 October 2021). To be more confident that the sequence taken will include the full promoter, we used 1.5–1.6 kb instead of 1.2 kb. Presumably, this region includes the core promoter (located ~40 bp upstream of the transcriptional initiation site), the proximal promoter (200–300 bp upstream of the core promoter), and the distal promoter (the rest of the fragment), where the proximal and distal regions contain various regulatory sequences, such as enhancers, silencers, insulators, and *cis*-elements, which contribute to the fine regulation of gene transcription.

**Figure 6:**
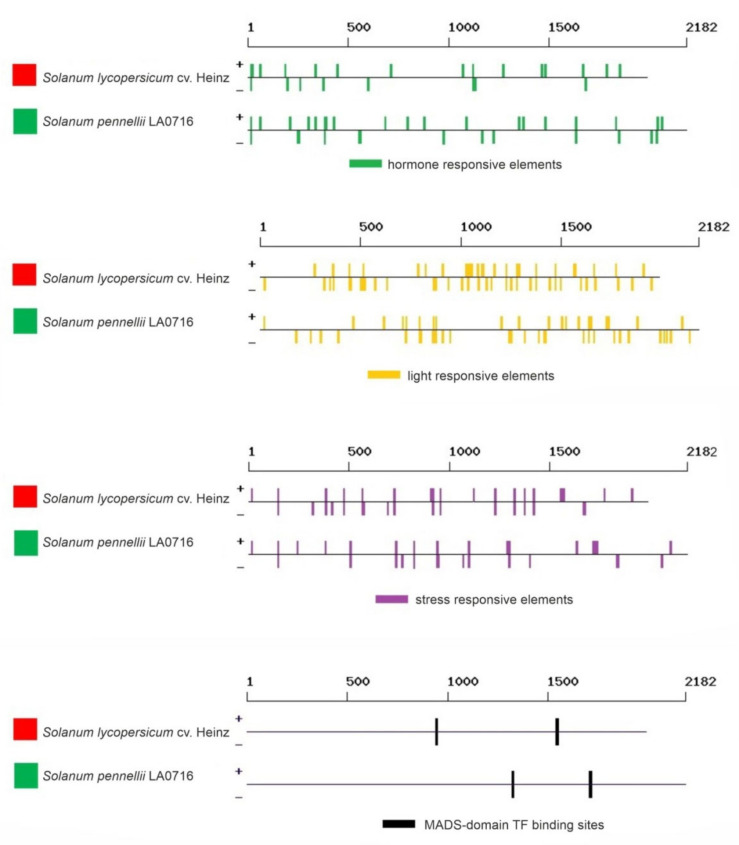
Regulatory elements identified in silico in the promoter sequences of *S. lycopersicum* cv. Heinz 1706 (FSCV) and *S. pennellii* LA0716 *Z-ISO* genes. Red and green squares correspond to the color of the fruit of the analyzed tomato accessions. The localization of the elements is rendered according to the data in Supplementary Table S2.

The search showed that the regulatory regions of the *Z-ISO* genes contained 131 regulatory elements, including 18 light-responsive (TCCC-motif, chs-CMA2a, TCT-motif, GT1CONSENSUS, chs-CMA1a, INRNTPSADB, GATABOX, Box 4, I-box, IBOXCORE, GT1-motif, G-box, TBOXATGAPB, REALPHALGLHCB21, ACGTATERD1, ASF1MOTIFCAMV, EBOXBNNAPA, and SORLREP3AT), 30 hormone-responsive (ABRE, ABRE3a, ABRE4, ABRELATERD1, ABRERATCAL, ERE, EECCRCAH1, ERELEE4, TGA-element, ASF1MOTIFCAMV, SURECOREATSULTR11, NTBBF1ARROLB, ARFAT, CATATGGMSAUR, TGA-box, CGTCA-motif, CPBCSPOR, GAREAT, PYRIMIDINEBOXOSRAMY1A, T/GBOXATPIN2, CAREOSREP1, DPBFCOREDCDC3, ARR1AT, MYBPLANT, MYBGAHV, AMYBOX2, RYREPEATBNNAPA, HEXMOTIFTAH3H4, AUXRETGA1GMGH3, and TCA-element), and 18 stress-responsive (STRE, LTRECOREATCOR15, SEBFCONSSTPR10A, GT1GMSCAM4, ARE, ANAERO3CONSENSUS, AS-1, CURECORECR, PREATPRODH, WRKY71OS, WBOXNTERF3, WBOXNTCHN48, MYB1AT, EBOXBNNAPA, MYCATERD1, BIHD1OS, BOXIINTPATPB, and BOXLCOREDCPAL). The other elements represented biding sites for transcription factors (TFs) (WRKY, MYB, ARR1, DOF, RAV1, bZIP, MADS, etc.), core promoter elements, elements with different predicted functions, and those with an unknown role (Supplementary Table S2, Figure 6). 

Among the identified regulatory elements, 31 (including 3 light-, 5 hormone-, and 2 stress-responsive) were absent in the promoter of *S. lycopersicum Z-ISO* and 18 (including 1 light- and 1 auxin-responsive) were absent in the promoter of *S. pennellii Z-ISO* (Figure 6). The numbers of eight elements significantly differed between *S. lycopersicum* and *S. pennellii* genes. Thus, ethylene-responsive EECCRCAH1, common promoter element CCAATBOX1, WRKY-binding sites WBOXATNPR1 and WBOXNTCHN48, ARR1-binding site ARR1AT, and polyadenylation signal POLASIG1 were over-represented in *S. pennellii*, whereas SORLREP3AT and bZIP-binding site DPBFCOREDCDC3 were over-represented in *S. lycopersicum* (Supplementary Table S2). 

### 2.4. Z-ISO Gene Expression in Tomato Cultivars and Wild Species

**Figure 7:**
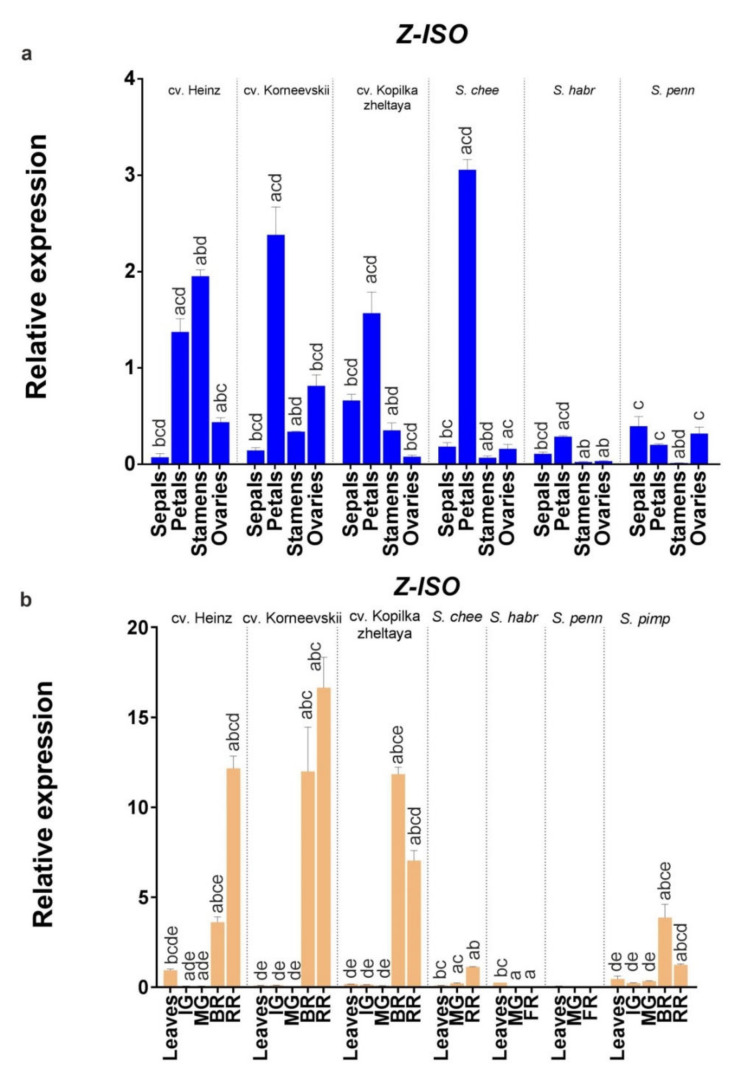
Comparison of *Z-ISO* mRNA expression in cultivars and wild species. (**a**) Flower organs; (**b**) leaves and fruit (IG, MG, BR, and RR or fully ripe [FR] stages). *S. lycopersicum* cultivars (Heinz, Korneevskii, and Kopilka zheltaya), *S. cheesmaniae* LA0421, *S. habrochaites* LA2144, and *S. pennellii* LA0716 were analyzed. The data were normalized to *Expressed* and *Actin* mRNA levels and presented as the mean ± SE (*n* = 3). Different low-case letters above the bars indicate statistically significant differences (*p* < 0.005) between gene expression levels in the tissues of one sample.

An analysis of *Z-ISO* expression in leaves, fruit (immature green, mature green, breaker, and fully ripe/red ripe stages), and flower organs (sepals, petals, stamens, and pistils) of *S. lycopersicum* (cv. Heinz, Korneevskii, and Kopilka zheltaya) and wild tomato species (YF *S. cheesmaniae* LA0421 and GF *S. habrochaites* LA2144 and *S. pennellii* LA0716) revealed that the expression of *Z-ISO* was accession-dependent (Figure 7). Thus, *Z-ISO* transcription was significantly higher in yellow petals of YF/RF species compared to GF species (by 8–12 times; Figure 7a) and in fully ripe fruit of YF/RF cultivars compared to YF wild species (by 7–17 times; Figure 7b). In fully ripe fruit, *Z-ISO* transcripts were present only in YF/RF accessions and absent in GF species (Figure 7b). *Z-ISO* transcription in breaker and fully ripe fruit stages had different dynamics, increasing with ripening in RF cultivars and YF *S. cheesmaniae* and decreasing in YF cv. Kopilka zheltaya (Figure 7b). *Z-ISO* mRNA expression was similar in the leaves of the analyzed accessions (Figure 7b).

### 2.5. Carotenoid and Chloropohyll Content in the Fruit of Tomato Cultivars and Wild Species 

**Figure 8:**
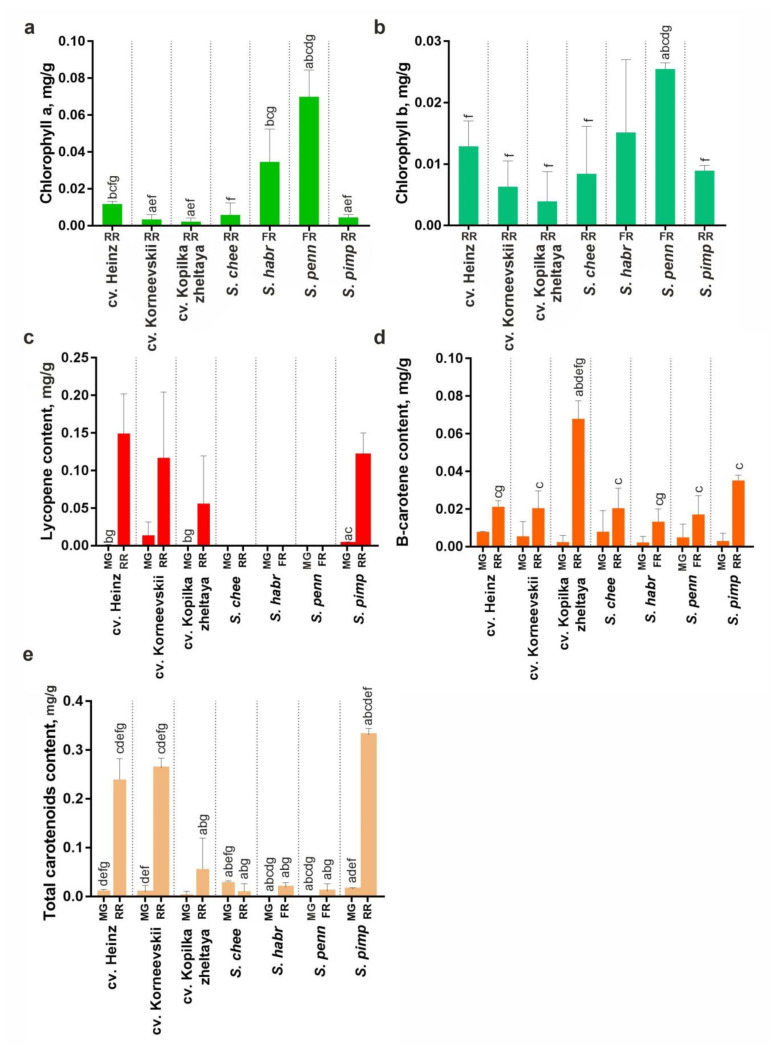
Chlorophyll (**a**,**b**) and carotenoid (**c**–**e**) content in MG and ripe fruit (RR in YF/RF accessions, FR in GF accessions) of *S. lycopersicum* (cv. Heinz, Korneevskii, Kopilka zheltaya), *S. cheesmaniae* LA0421 (*S. chee*), *S. habrochaites* LA2144 (*S. habr*), *S. pennellii* LA0716 (*S. penn*), and *S. pimpinellifolium* VIR1018 (*S. pimp*). Low-case letters above the bars indicate statistically significant differences (*p* < 0.005) between pigment content in the same tissue of different samples.

The accumulation of total (xanthophylls + carotenes) and specific (lycopene and β-carotene) carotenoids in mature green and ripe fruit and that of chlorophyll (*a* and *b*) in ripe fruit was assessed in RF *S. lycopersicum* (cv. Heinz, Korneevskii, and Kopilka zheltaya) and *S. pimpinellifolium* (VIR1018), YF *S. cheesmaniae* (LA0421), and GF *S. habrochaites* (LA2144) and *S. pennellii* (LA0716). The results indicated that during fruit ripening, the carotenoid content increased in all analyzed accessions, except YF cv. Kopilka zheltaya and *S. cheesmaniae* where the total amount of carotenoids changed insignificantly. The fruit of wild YF and GF tomatoes lacked lycopene and contained 25–30 times less total carotenoids than those of *S. lycopersicum* cultivars and *S. pimpinellifolium.* Fruit β-carotene content was almost the same for all analyzed accessions (Figure 8c,d). In ripe fruit, the total chlorophyll content was higher for GF *S. pennellii* and *S. habrochaites* compared to YF/RF accessions (Figure 8a,b).

## 3. Discussion 

Carotenoids are a group of yellow, orange, and red pigments synthesized by plants, algae, fungi, and photosynthetic bacteria. Carotenogenesis is closely associated with the emergence of photosynthetic organisms, and is thought to have evolved through recruitment of genes from noncarotenogenic bacteria [7]. This conclusion is based on comparative genomics data, including analysis of the *Z-ISO* gene across species from cyanobacteria to angiosperms in which it probably evolved from an ancestral gene related to the bacterial *NnrU* required for denitrification [7].

Carotenoids act as color attractants, photo-protectors, antioxidants, and precursors of phytohormones such as ABA and strigolactones [1]. Carotenoids are synthesized de novo on plastid membranes in all plant organs, where they can be visually detected by their color. During ripening of the cultivated tomato (*S. lycopersicum*) fruit, chloroplasts are replaced by chromoplasts, which accumulate carotenoids; as a result, the fruit color changes from green to yellow, orange, or red depending on the ratio of carotenoid types [3,4,18,19]. The wide variety of fruit colors in tomato cultivars made *S. lycopersicum* an excellent model to study carotenoid biosynthesis and accumulation in fleshy fruit. Furthermore, *S. lycopersicum* has 12 wild relatives also belonging to *Solanum* section Lycopersicon, which differ in the color of ripe fruit. Among them, *S. pimpinellifolium* is the most evolutionarily recent and forms red fruit, whereas the more distant *S. cheesmaniae* and *S. galapagense* have yellow to orange fruit, and other more ancient species produce only green fruit [20,21]. 

In the present study, we identified and characterized *Z-ISO* gene homologs in12 *S. lycopersicum* cultivars and five wild tomato species (*S. pimpinellifolium*, *S. cheesmaniae*, *S. chilense*, *S. habrochaites*, and *S. pennellii*) differing in ripe fruit color (Table 1). The genes did not have paralogous copies in the genome and were orthologs of the *Z-ISO* genes of *A. thaliana* (Q9SAC0), *Z. mays* (B4FHU1), and *O. sativa* (NP 001066625.1), suggesting high functional conservation of *Z-ISO* among plant species [5,6,7,8,14,15,17,22]. Therefore, Z-ISO homologs in wild and cultivated tomato species are likely to have enzymatic activity and be functionally integrated in the carotenoid biosynthetic pathway by performing *cis*-*trans* isomerization of 9,15,9′-tri-*cis*-ζ-carotene, suggesting Z-ISO as a key enzyme that determines the switch to the synthesis of colored carotenoids and, as a result, ripe fruit color in tomato species. The same yellow petal color in the flowers of wild and cultivated tomato species [23] suggests that in this case, carotenoid biosynthesis and accumulation did not undergo significant evolutionary changes, whereas such changes must have taken place in the fruit, where *Z-ISO* enzymatic activity is likely to contribute to the color differences. Indeed, the phylogeny of plant species based on Z-ISO protein sequence not only corresponds to the generally accepted evolution history of Solanaceae [24], but also allows us to divide the analyzed tomatoes into clades of RF/YF and GF species (Figure 4), indicating that Z-ISO may be a good phylogenetic marker to study the evolution of tomatoes. The levels of *Z-ISO* expression and/or enzymatic activity may be markers of lycopene and carotene accumulation in the fruit of RF/YF tomato species and the lack of it in those of GF species. 

Despite the differences in fruit color, the identified Z-ISO homologs were structurally conserved in all analyzed tomato accessions (Figure 1, Figure 2 and Figure 3). The predicted presence of an N-terminal transit peptide, transmembrane helices, and a mature protein corresponding to the Z-ISO enzyme suggests that the *Z-ISO* homologs have isomerization activity and are integrated into the membranes of plastids, i.e., chloroplasts in photosynthetic tissues and chromoplasts in sink organs such as fruit. The transit peptide, which is not required for the enzymatic activity of Z-ISO [17], did not contain radical aa substitutions (Figure 2) but was the most polymorphic Z-ISO region, distinguishing the *Solanum*, *Capsicum*, and *Lycium* genera from the other plant species (Figure 3). In the course of evolution, the only motif at the N-terminus of the transit peptide that was conserved in all analyzed accessions including diatoms was MASSJFLSHPA (Figure 3), which may emphasize a key role of this consensus in Z-ISO protein relocation to the plastid membrane. Residues H153, H269, D297, and C266, as well as the last seven C-terminal residues essential for Z-ISO activity [17,25] were conserved in all analyzed accessions (Supplementary Figure S3). The detected radical substitutions H288P, H211Q, and H194P (Figure 2) should not affect the activity of Z-ISO in tomato, since His residues (except for H153 and H269) are not required for Z-ISO function [17]. Altogether, these data suggest a highly conserved role of this enzyme in wild and cultivated tomato species with differently colored ripe fruit.

Considering the conserved structure of Z-ISO, the difference in fruit color may be due to differential regulation of *Z-ISO* expression. Thus, it has been shown that high *Z-ISO* expression corresponds to the increased content of total carotenoids and α-/β-carotenes in *Chelidonium majus* flowers and leaves [22], whereas its low expression leads to noticeable lightening of sweet orange (*Citrus sinensis*) pigmentation [26], and the absence of *Z-ISO* expression correlates with the lack of colored carotenoids and accumulation of colorless *cis*-carotenes [5,7,8,10,15,16]. Consistent with these findings, we observed that the level of *Z-ISO* expression positively correlated with the carotenoid content in tomato fruit (Figure 7 and Figure 8). At the same time, the difference in the level of gene expression in flower petals does not visually affect their color; petals, regardless of the tomato sample, are yellow due to the accumulation of mainly xanthophylls in petal chromoplasts [23].

Species-specific differences in Z-ISO expression may be due to variations in the promoter and 5′-UTR sequences, as evidenced by considerable variability in the regulatory regions of GF tomato species, in which it was 1.38–1.58 times higher than in cultivars (Table 2). We observed high variability of *Z-ISO* promoter sequences compared to cv. Heinz not only in wild GF species (9.38%) but also in cultivars (6.81%) (Table 2). At the same time, it has been reported that in 12 *S. lycopersicum* accessions the promoters of the other genes in the carotenoid biosynthetic pathway (*GGPPS1*, *PSY2*, *PDS*, *ZDS*, *PSY1*, *CrtISO*, *LYC-b*, and *LYC-e*) have 97–100% sequence identity [27]. These data may indicate a key role of Z-ISO in carotenoid biosynthesis. 

Several studies have investigated transcriptional regulation of the genes involved in carotenoid biosynthesis in tomato. Thus, it has been shown that in *S. lycopersicum* and *S. pennellii* the upstream regions of the *PSY1* gene contain at least 37 types of regulatory elements, indicating that *PSY1* transcription may be altered in response to light, abiotic stresses, and hormones [28,29]. Our analysis revealed that the upstream regions of *Z-ISO* also contained *cis*-elements associated with response to light, hormones, and stresses and did not significantly differ between RF *S. lycopersicum* cv. Heinz and GF *S. pennellii* LA0716 (Figure 6, Supplementary Table S2), indicating high degree of conservation in the mechanism regulating *Z-ISO* transcription. The promoters of *PSY1*, *CrtISO*, and *LYC-b* genes contain binding sites for MADS-domain TFs [27], and it has been shown that MADS-RIN directly regulates *PSY1*, *Z-ISO*, and *CrtISO* expression through binding to their promoters [30,31]. We also detected two MADS-TF-binding sites in the *Z-ISO* promoter of both *S. pennellii* LA0716 and *S. lycopersicum* cv. Heinz (Supplementary Table S2). At the same time, the *Z-ISO* promoter of *S. pennellii* contained significantly fewer MYB- and bZIP TF-binding sites but more WRKY- and ARR1 TF-binding sites than that of *S. lycopersicum* cv. Heinz; in addition, the two promoters differed by the presence of multiple regulatory elements with unknown or poorly characterized function (Supplementary Table S2). Such dissimilarity in the promoter structures may account for the differences in *Z-ISO* expression levels between GF and RF tomatoes. Of interest is the excess of ethylene-sensitive elements in the *S. pennellii Z-ISO* promoter. Ethylene plays a key role in the ripening of climacteric fruits and in regulating plant growth and development [32]. In addition, ethylene is involved in plant responses to various biotic and abiotic stresses [33]. Thus, the presence of a lower number of such elements in the *Z-ISO* promoter of cultivated tomato compared to wild *S. pennellii Z-ISO* may reflect the increased ability of the latter to respond to stressful growth conditions, including during fruit ripening.

The conversion of chloroplasts to chromoplasts, which store the carotenoid pigments, is essential for fruit ripening process in tomato and is the primary factor in tomato fruit coloring [34,35]. The other factor is the expression of PSY1, which catalyzes the first stage of carotenoid biosynthesis and defines the content of colored carotenoids [29]. In this scenario, Z-ISO, which determines the switch from the production of colorless carotene to that of colored carotenoids, should contribute to the difference in the color of ripe fruit between YF/RF and GF tomato species. 

## 4. Materials and Methods

### 4.1. Plant Material

Accessions of wild tomato species (*Solanum* section Lycopersicon) were kindly provided by the Tomato Genetics Resource Center (Davis, CA, USA; https://tgrc.ucdavis.edu/, accessed on 20 January 2021) and NI Vavilov Institute of Plant Genetic Resources (VIR, St-Petersburg, Russia) (Table 1). *S. lycopersicum* cultivars were kindly provided by the Federal Scientific Center of Vegetables (FSCV, Moscow region, Russia) (Table 1). Plants were grown in a greenhouse with temperature kept at 28 °C/23 °C during a 16-h/8-h day/night light cycle (light intensity, 300–400 μmol m^−2^ s^−1^). Young leaves, opened flower parts (sepals, petals, stamens, and pistils), and fruit (at IG, MG, BR, and RR/fully ripe [FR] stages) were collected in two biological replicates and homogenized in liquid nitrogen. The MG stage was defined as firm green fruit of a final (maximal) size. Ripe fruit in GF species were defined by softness and in YF/FR species—by color due to chlorophyll degradation and carotenoid accumulation [36,37,38].

### 4.2. Identification and Structural Characterization of Tomato Z-ISO Genes

To amplify full-length *Z-ISO* genes from tomato accessions, gene-specific primers (Supplementary Table S3) were designed based on *Z-ISO* genomic sequences of *S. lycopersicum* cv. Heinz 1706 (Gene ID: 101253762, NC_ NC_015449.3 [67104216… 67112934], http://www.ncbi.nlm.nih.gov/Genbank, accessed on 15 May 2021, genome annotation releases; Sol Genomics Network (Solyc12g098710.1, https://solgenomics.net/, accessed on 15 May 2021)), *S. pimpinellifolium* (NCBI ID: 11283; assembly ASM1496433v1), *S. habrochaites* (NCBI ID: 24151; assembly Sohab10), *S. pennellii* (NCBI ID: 24150; assembly SPENNV200), and *S. arcanum* (NCBI ID: 31602; assembly Soarc10). Manual revision of sequence polymorphisms and additional evaluation were performed using Primer3 (http://frodo.wi.mit.edu/primer3/, accessed on 10 June 2021).

Genomic DNA was isolated from young leaves of a single plant of each accession as previously described [39] with additional double deproteinization with phenol-chloroform and used as a template (100 ng) for PCR amplification at the following conditions: initial denaturation at 95 °C for 10 min, 35 cycles of denaturation at 95 °C for 30 s, primer annealing at 57 °C for 30 s, and extension at 65 °C for 4 min, and final extension at 65 °C for 10 min. The amplified PCR products of the expected size were purified by using the QIAEX^®^ II Gel Extraction kit (QIAGEN, Hilden, Germany), cloned in the pGEM^®^-T Easy vector (Promega, Madison, WI, USA), and sequenced (3–5 clones for each accession) on ABI Prism 3730 DNA Sequencer (Applied Biosystems, Waltham, MA, USA) using the designed primers (Supplementary Table S3). 

Multiple sequence alignments and structural and phylogenetic analyses of *Z-ISO* genes and the encoded proteins were conducted with MEGA 7.0 [40]. Predicted proteins were characterized by conserved domains, sites, spatial structure, and motifs using web-based tools (NCBI-CDD, https://www.ncbi.nlm.nih.gov/cdd, accessed on 20 July 2021; UniProt, https://www.uniprot.org/, accessed on 10 July 2021; Phyre2, http://www.sbg.bio.ic.ac.uk/phyre2/, accessed on 25 July 2021 [41], and MEME 5.4.1 [42], http://meme-suite.org/tools/meme, accessed on 24 September 2021) and the functional importance of residue substitutions (PROVEAN; [43]). The presence of signal peptides was analyzed using SignalP 5.0 (http://www.cbs.dtu.dk/services/SignalP/, accessed on 10 July 2021) and ChloroP (http://www.cbs.dtu.dk/services/ChloroP/, accessed on 10 July 2021). A phylogenetic dendrogram was constructed based on protein sequences using maximum likelihood method and Jones-Taylor-Thornton (JTT) with Gamma distributed (G = 2) model in MEGA 7.0.26; confidence for tree topologies was estimated by bootstrap values of 1000 replicates.

For comparative structural analysis, the complete sequences of Z-ISO homologs in different plants were extracted from the NCBI database (https://www.ncbi.nlm.nih.gov/, accessed on 19 October 2021) (Supplementary Table S1). 

### 4.3. In Silico Analysis of mRNA Expression 

The expression of *Z-ISO* homologous genes in tomato tissues was determined based on RNA-seq data for *S. lycopersicum* (cv. Heinz, Micro-Tom, M82, Moneymaker, SUN1642, and Alisa Craig) and *S. pimpinellifolium* LA1589 (TomExpress, http://tomexpress.toulouse.inra.fr/, accessed on 10 August 2021; Tomato Expression Atlas, https://tea.solgenomics.net, accessed on 10 August 2021).

### 4.4. RNA Extraction and Quantitative Real-Time PCR (RT-qPCR) 

Tissues (0.5 g) of young leaves, flower buds, yellow petals, and fruit (IG, MG, BR, and FR stages) were ground to powder in liquid nitrogen. Total RNA was isolated from samples using the RNeasy Plant Mini Kit (QIAGEN, Hilden, Germany), admixtures of genomic DNA were removed (RNase free DNasy set; QIAGEN, Hilden, Germany). RNA preps were qualified by gel electrophoresis, and used for first-strand cDNA synthesis (Reverse Transcription System; Promega, Madison, WI, USA) with an oligo-dT primer. RNA and cDNA concentrations were quantified by fluorimetry (Qubit^®^ Fluorometer, Thermo Fisher Scientific, Waltham, MA, USA). Quantitative RT-PCR was performed in a CFX96 Real-Time PCR Detection System (Bio-Rad Laboratories, Hercules, CA, USA) with 2.5 ng of cDNA, SYBR Green RT-PCR mixture (Syntol, Moscow, Russia), and specific primers (Supplementary Table S3) at the following cycling conditions: initial denaturation at 95 °C for 5 min and 40 cycles of denaturation at 95 °C for 15 s and annealing/extension at 60 °C for 40 s. To normalize the levels of *Z-ISO* expression, two reference tomato genes, *Expressed* (SGN-U346908) and *Actin 2/7* (NM_001330119.1) [44,45], were used.

The qRT-PCR results were statistically analyzed with Graph Pad Prism version 7.02 (GraphPad Software Inc., San Diego, CA, USA; https://www.graphpad.com/scientific-software/prism/, accessed on 20 July 2021) and the data were expressed as the mean ± standard deviation (SE) based on three technical replicates of two biological replicates. The unequal variance (Welch’s) *t*-test was applied to assess statistical significance of differences in gene expression between tissues within the same species and between the same tissues of different tomato species; *p* < 0.05 was considered to indicate statistical significance.

### 4.5. Promoter and 5′-UTR Identification and Analysis

The regulatory regions of *Z-ISO* genes were amplified by PCR using specific primers designed based on *S. lycopersicum* cv. Heinz 1706 genome sequence (Supplementary Table S3), purified, cloned, and sequenced. The search for specific *cis*-elements in promoters and 5′-UTRs (~2.0 kb regions upstream of the initiation codon) was performed using the PlantCARE database, which provides evaluation of *cis*-regulatory elements, enhancers, and repressors (http://bioinformatics.psb.ugent.be/webtools/plantcare/html/; accessed on 25 August 2021; [46]). 

### 4.6. Carotenoid and Chlorophyll Content

Total carotenoid content was measured by spectrophotometry in two biological and three technical replicates using a modified Folch method [47,48]. Briefly, 0.2 g of plant tissue was homogenized in Folch solution (2:1 chloroform-methanol [*v*/*v*]) in the presence of trace amounts of Mg_2_CO_3_ [46], incubated at 4 °C for 1 h, and centrifuged at 4000 rpm for 10 min at 4 °C. The lower chloroform phase was collected, and lycopene, β-carotene, and total carotenoid contents (xanthophylls plus carotenes) were measured as previously described [49] using a spectrophotometer (Basic, Eppendorf, Hamburg, Germany) and calculated according to the published formulas [29].

## 5. Conclusions

We identified *Z-ISO* homologous genes in red-, yellow- and green-fruited accessions of wild tomato species (*Solanum* section Lycopersicon) and *S. lycopersicum* cultivars. Z-ISO homologs shared high similarity of structure, conserved motifs, and functionally important sites; however, *Z-ISO* promoter and 5′-UTR sequences of green- and red/yellow-fruited tomatoes differed in the numbers of light-, hormone- and stress-responsive elements, as well as in transcription factor-binding sites. *Z-ISO* expression positively correlated with carotenoid content in ripe tomato fruit. *Z-ISO* transcriptional profiling in cultivars and wild species suggests that the level of *Z-ISO* expression may be a key factor defining carotenoid accumulation in ripe tomato fruit. Our results provide valuable data for further functional and evolutionary characterization of carotenogenesis in fleshy fruit, and may be used for tomato biotechnology research, since Z-ISO affects the synthesis not only of carotenoids, but also of the hormonal end-products (strigolactones and ABA) of the carotenoid pathway. 

## Figures and Tables

**Table 1:** Characteristics of *Z-ISO* genes in tomato cultivars and wild species.

Species	Cultivar/Accession	Ripe Fruit Color	NCBI Accession Number	Gene/CDS (bp)	5′-UTR/Promoter (bp)	Protein (aa)
Extracted from the NCBI database						
*Solanum lycopersicum* L.	cv. Heinz 1706	Red	NC 015449.3: 67104216–67112934 chromosome 12 Solyc12g098710	4008/1110	438/1564	369
*Solanum pimpinellifolium* L.	LA0480		NRDK01042673.1: 47213–54353 Scaffold3068453	4009/1110	438/1459	369
*Solanum pennellii* Correll, 1958	LA0716	Green	Gene ID: CCXL01009615.1 (3669..9559)	4006/1110	468/1714	369
*Solanum habrochaites* S. Knapp & D. M. Spooner, 1999	var. *LYC4*	Green	Gene ID: CBYS010011028.1 (46212..52055)	4003/1110	473/1307	369
*Solanum arcanum* Peralta	LA2157	Green	Gene ID: CBYQ010012533.1 (26020..31886)	4006/1110	473/1648	369
Identified in this study						
*S. lycopersicum* L.	cv. Heinz (FSCV)	Red	OK318866	4007/1110	438/1549	369
	cv. Korneevskii	Red	OK318867	4006/1110	438/1551	369
	cv. Garmoshka	Red	OK318869	4009/1110	438/1562	369
	cv. Malinovyi silach	Red	OK318868	4004/1110	438/1561	369
	cv. Zemba	Red-Violet	OK318871	4010/1110	438/1560	369
	cv. Paul Robeson	Red-Violet	OK318870	4009/1110	436/1559	369
	cv. Black Jack	Red-Violet	OK318872	4008/1110	436/1560	369
	cv. Christmas blueberry	Red-Violet	OK318873	4009/1110	436/1567	369
	cv. Black cherry	Red-Violet	OK318874	4008/1110	438/1552	369
	cv. Cherry zhelto-oranzhevye	Yellow/Orange	OK318875	4008/1110	438/1548	369
	cv. Kopilka zheltaya	Yellow	OK318876	4008/1110	438/1548	369
	cv. Nesozrevayuschii Natasha (NN-25)	Pale Yellow	OK318877	4007/1110	437/1525	369
*S. pimpinellifolium* L.	VIR1018	Red	OK318858	4008/1110	438/1459	369
*Solanum cheesmaniae* (L. Riley) Fosberg, 1987	LA0421	Yellow/Orange	OK318859	4010/1110	363/1394	369
*Solanum chilense* (Dunal) Reiche	LA2884	Green	OK318865	3999/1110	413/1313	369
	LA1963	Green	OK318864	3994/1110	413/1303	369
*S. habrochaites* S. Knapp & D. M. Spooner, 1999	LA1777	Green	OK318860	3972/1110	474/1398	369
	LA2144	Green	OK318861	4003/1110	473/1293	369
*Solanum pennellii* Correll, 1958	LA0716	Green	OK318863	4006/1110	468/1714	369
	LA1926	Green	OK318862	4008/1110	468/1714	369

VIR—NI Vavilov Institute of Plant Genetic Resources (St-Petersburg, Russia); FSCV—Federal Scientific Center of Vegetables (Moscow region, Russia).

**Table 2:** Variability of *Z-ISO* genes in *S. lycopersicum* cultivars and wild tomato species.

Gene Region/Translated Product	Variability (%)				
	Cultivars *	Cultivars and Wild Species *	Wild RF/YF Species *	Wild GF Species *	Wild GF Species **
Promoter	6.81	17.14	3.30	9.38	7.71
5′-UTR	6.47	21.50	7.31	10.23	10.86
Gene	5.92	14.89	3.85	6.22	6.67
cDNA	3.96	10.36	1.44	5.14	5.50
Protein	7.03	16.80	2.70	9.19	8.65

GF—green-fruited; RF—red-fruited; YF—yellow-fruited. * vs. *S. lycopersicum* cv. Heinz 1706; ** vs. *S. arcanum* LA2157.

## Data Availability

The data on ID numbers of the identified genes are provided in Table 1.

## Supplementary Materials

The following are available online at https://www.mdpi.com/article/10.3390/plants10112365/s1. Figure S1: Carotenoid biosynthesis scheme; Figure S2: Alignment of the *Z-ISO* gene from *Solanum lycopersicum* cultivars and wild tomato species (*Solanum* section Lycopersicon); Figure S3: Alignment of the Z-ISO protein sequence from *S. lycopersicum* cultivars and wild tomato species (*Solanum* section Lycopersicon); Figure S4: Spatial structure of the Z-ISO protein from *S. lycopersicum* cv. Heinz: (a) 3D model, (b) secondary structure, and (c) transmembrane helix prediction; Table S1: Z-ISO homologs from different plant species used for MEME and phylogeny analysis; Table S2: Regulatory elements found in the promoter and 5′-UTR of the *Z-ISO* gene from *S. lycopersicum* cv. Heinz and *Solanum pennellii* LA0716; Table S3: List of primers used for *Z-ISO* gene amplification, sequencing, and expression analysis.
